# Diachronic trends of employment outcome of prevocational training in psychiatric rehabilitation

**DOI:** 10.1186/1744-859X-9-2

**Published:** 2010-01-06

**Authors:** Rossetos J Gournellis, Eugenia S Triantafillou, Michael G Madianos, Niki N Tsinia, Dimitris N Ploumpidis, Vlassis D Tomaras

**Affiliations:** 1 Second Department of Psychiatry, University of Athens, Medical School, Attikon General Hospital, Athens, Greece; 2 First Department of Psychiatry, University of Athens, Medical School, Eginition Hospital, Athens, Greece; 3 University of Athens, School of Health Sciences, Faculty of Nursing, Community Mental Health Centre, Zografou, Athens, Greece

## Abstract

**Background:**

Although many rehabilitation programmes of prevocational training for chronic mentally ill persons living in the community have been funded, there is scarce literature about the diachronic trends of their long-term employment outcome. Thus the aim of the present study was to compare the 2-year employment outcome of three groups of chronic psychiatric outpatients, having attended similar prevocational rehabilitation programmes in different periods of time.

**Methods:**

The first group (1984 to 1986) comprised 67 rehabilitees, the second (1988 to 1989) 53 rehabilitees and the third (2000 to 2001) 56 rehabilitees. The three groups were compared with regard to employment follow-up achievements and hospitalisation rates assessed at the end of the 2-year follow-up period by a constructed overall index, encompassing employment qualitative and quantitative characteristics.

**Results:**

The third group compared to the first and second ones presented a worse employment outcome. No differences were found among the three groups with regard to hospitalisation rates.

**Conclusions:**

There has been a decline in the employment outcome of prevocational training during the current decade. This decline can be attributed to contextual adverse factors such as unemployment, a more demanding labour market and disability allowances offered by the state (the 'benefit trap'). Moreover, the training itself may be 'old-fashioned' enough, thus providing the trainees with inadequate skills to obtain and maintain a competitive job.

## Background

Work can be seen as an essential societal and personal value, and in this context preparation for employment (or re-employment) has become an integral component of psychosocial rehabilitation programs. It is reported that unemployment rates for people with long-term psychiatric disorders were extremely high (88%) during the 1990s [1]. Furthermore, Perkins and Rinaldi [2], comparing the unemployment rates of mentally ill persons with local employment rates in a borough of London throughout the same decade, found that the former continued to increase (from 80.3% to 91.9%) while the latter tended to decrease (from 13.8% to 4.9%). Provided that at the same period the unemployment rates for disabled people remained stable (around 60%) and that the unemployment rates were greatest among those diagnosed as schizophrenics, the authors proposed discrimination against the mentally ill as the strongest, among others, explanation for their findings.

Psychiatric rehabilitation services strive to reverse the gloomy perspective of employment for persons with psychiatric disabilities. Meta-analyses of controlled trials investigating the employment outcomes of occupational rehabilitation interventions indicate that such interventions are quite beneficial: up to 58% of the participants gained competitive employment [3,4]. Yet, it has been noticed that job tenure is often short [5].

Prevocational training assumes that a period of preparation is needed before entering competitive employment. It includes training on work-related skills in sheltered workshops, social skills training, transitional employment (the rehabilitees work in a job that is 'owned' by the rehabilitation agency for a certain period), work crews and other activities, preparing the trainees for a full-time or part-time competitive job [6]. The issue of whether the various approaches of prevocational rehabilitation such as the 'threshold' programmes, industrial workshops, employment preparation, counselling, transitional work, and so on, compared to standard care, achieve better results with respect to employment outcome still remains unclear [7-11]. The best results, in terms of paid, competitive employment, is claimed to be obtained by 'supported employment', which is the type of occupational intervention oriented to rapid job search and placement with ongoing and on-site job support, counselling and problem solving, instead of the traditional prolonged and stepwise prevocational and/or vocational training [12]. In particular, systematic reviews and meta-analyses have shown that at 12 months only 12% of prevocational training trainees were employed, instead of 34% of 'supported employment' participants [3,4].

However, the supported employment scheme has been tested almost only in the USA, and it is of uncertain generalisability in countries with dissimilar economic structures. The conventional prevocational training still dominates in Europe; even in the USA, at least until recently, 3,000 'psychiatric rehabilitation providers' were offering different forms of it. In the UK as well, prevocational training appears to be still the norm [4,13]. Nevertheless, the trends in vocational status of people who received prevocational training while experiencing chronic mental problems remain still unclear.

Therefore the aim of this study was to investigate the diachronic trends of employment, following the prevocational rehabilitation intervention provided at different spans in a European capital (Athens, Greece).

## Methods

All subjects were chronic psychiatric outpatients who attended and completed a community-based prevocational rehabilitation training in a network run by the First Department of Psychiatry, University of Athens, during different periods of time. The first group (A) comprised 69 trainees who completed a 12-month training programme during the years 1984 to 1986 and originated from a group of 76 trainees (7 dropouts during attendance). The second group (B) comprised 65 trainees who completed a training of the same duration in the years 1988 to 1989 and originated from a group of 75 (10 dropouts). The third group (C) comprised 58 trainees who completed a 14-month training during the years 2000 to 2001 and originated from a group of 63 (5 dropouts).

All members of the three groups shared common characteristics: they fell into the age range 19 to 45, they had been symptomatic for at least 2 years, they were in remission at their entry to the programme and they were under neuroleptic medication. No participants suffered from organic mental disorder, severe developmental disorder and/or comorbid substance abuse or misuse. Regarding diagnosis, 60 subjects (87%) of group A and 39 (67.2%) of group C were experiencing schizophrenia or schizoaffective disorder. The remaining participants were mostly assigned to severe obsessive-compulsive disorder (OCD) and bipolar disorder diagnoses. In contrast to groups A and C, group B was an experimental sample [14] consisting exclusively of patients experiencing schizophrenia. Diagnosis was put at intake to the rehabilitation unit according to Diagnostic and Statistical Manual of Mental Disorders (DSM) criteria: DSM-III [15] for group A, DSM-III-R [16] for group B and DSM-IV [17] for group C.

The three group participants received a similar training and treatment intervention programme. A prolonged form of prevocational training was delivered in various workshops, namely PCs, wood curving, bamboo, bookbinding, leather craft, seal making and agriculture, on a 5-h daily schedule and they were paid the minimum wages of an unskilled worker. Emphasis was put on punctuality, acceptance of workshop discipline, cooperation with workmates and staff, and achievement of reasonable standard of finished work. The principles of practice included praise, feedback and instruction. Prevocational training was accompanied by psychosocial therapeutic intervention on individual and group basis enhancing destigmatisation, self-esteem and social skills. During these sessions patients were prompted to express their feelings and interests and to recognise the emotions and attitudes of others. Compliance with medication was one of the key focus points of attention of the therapeutic team. Family intervention comprised 12 monthly group sessions for relatives, each of them lasting 90 min. These sessions included psychoeducation and support for the family members in order to reduce criticism of the patient and relieve the carer's burden (excluding for controls of the trial based on group B [14]).

### Assessments

The discharge assessment is considered as the baseline assessment here. The assessment criteria used at discharge for each group were not similar; the only common instrument being the Global Assessment Scale/Global Assessment of Functioning (GAS/GAF) scale [18,19].

After discharge, all rehabilitees were followed-up for 2 years. Every 6 months data were collected concerning their clinical and employment status. Hospitalisation was defined as an admission to a psychiatric ward for at least 2 days. Relapse, though defined, failed to be recorded, due to inconsistencies in the gathered information. Employment status incorporates both quantitative (for example, duration, full-time vs part-time work) and qualitative (for example, competitive work in the open job market, subsidised work, sheltered employment) features. Thus it was decided to construct an index combining both dimensions (employment index (EMI)) (Table 1) in order to measure the employment outcome of our rehabilitees. For purposes of statistical analysis we transformed the initially four-item scale (available from the authors) to a categorical one consisting of: (a) low employment outcome, incorporating the items poor and rather poor, and (b) fair employment outcome, incorporating the items of intermediate and rather satisfactory (see Table 1).

**Table 1:** Employment index (EMI) combining type and maintenance of employment throughout the 2-year follow-up

Category of employment outcome	Salaried full-time employment (months)	Salaried part-time employment (months)	Subcontract/homework employment (months)	Sheltered employment (months)
1 = Low	<4	<6	<8	Yes
2 = Fair	≥ 4	≥ 6	≥ 8	-

Dropouts occurred throughout the 2-year follow-up period: 2 subjects in group A, 12 in group B and 2 in group C. Thus the final sample sizes were reduced to 67, 53 and 56, respectively. Dropouts were excluded from statistical analysis. In particular, for group B, where the dropout rate was considerable, the 12 dropouts compared to the 53 participants who completed the programme were not found to differ in any demographic and/or clinical characteristic (age, years of education, length of illness, number of hospitalisations and GAS score by one-way analysis of variance (ANOVA)).

### Statistical analysis

For comparisons among the three groups with regard to categorical variables a two-tailed Pearson χ^2 ^test was used and for continuous variables with normal distribution as well as for pairwise comparisons between the groups when the overall *F *test indicated that the subgroups means were significantly different we used a two-tailed one-way ANOVA with *post hoc *comparisons. In addition, Bonferroni correction was applied in order to account for multiple comparisons (new level required of statistical significance *P *= 0.002). Lastly, in order to measure the magnitude of the training effect in each group we used the Cohen's *d*. An effect size from 0.00 to 0.32 was rated as 'small', from 0.33 to 0.55 as 'medium' and greater than 0.55 as 'large' [20].

## Results

### Baseline assessment

All groups were predominately male: 43 (64.2%) in group A, 39 (73.6%) in group B and 36 (64.3%) in group C, respectively (multiple χ^2 ^test). In a preliminary analysis, we compared the groups along demographic and clinical characteristics at discharge. As seen in Table 2, the average number of years of education for subjects was around 11 years. The members of groups A and B were significantly younger as compared to the members of group C (*F *= 18, df = 2, *p *= 0.000). Although young, the members of all groups were chronic patients with a mean duration of their disorder 7.2 (SD 3.9), 7.9 (SD 5.1) and 14.2 (SD 7.8) years, respectively. Group C was significantly more chronic than groups A and B (*F *= 18, df = 2, *p *= 0.000). Group B presented at discharge the worst total GAS/GAF score in comparison to groups A and C (*F *= 11.3, df = 2, *p *= 0.000).

**Table 2:** Demographic and clinical characteristics of the three groups A to C (baseline assessment)

Variable	Groups						Analysis			
		
	A (N = 67), (%)		B (N = 53), (%)		C (N = 56), (%)		Test value	df	*p *value	Significant pairwise comparisons
Sex (male/female)	43/24 (64%/36%)		39/14 (73%/27%)		36/20 (64%/36%)		1.4^a^	2	NS	
	Mean	SD	Mean	SD	Mean	SD				
Age	28.3	6.0	28.5	5.5	34.9	8.1	18.0^b^	2	0.000	C > A and B
Years of education	10.9	3.1	11.5	2.6	11.0	3.1	0.6^b^	2	NS	
Length of illness (years)	7.2	3.9	7.9	5.1	14.2	7.8	25.6^b^	2	0.000	C > A and B
GAS/GAF at discharge	59.7	10.6	51.0	10.6	58.6	10.0	11.3^b^	2	0.000	B < A and C

^a^Multiple χ^2^; ^b^one-way analysis of variance (ANOVA).

df = degrees of freedom; GAS/GAF = Global Assessment Scale/Global Assessment of Functioning; NS = not significant.

### Follow-up assessment

The employment follow-up outcome for the rehabilitees, assessed by the constructed overall EMI, was as follows: 30 (44.8%) members of group A presented low outcome, and the rest (37 (55.2%)) fair. In group B, 31 (58.5%) members presented low employment outcome and 22 (41.5%) fair. In group C, 49 (87.5%) members were classified as low, and 7 (12.5%) as fair on employment outcome (Table 3).

**Table 3:** Comparisons of the three groups A to C with regard to their 2-year follow-up employment outcome

Category of employment outcome	Groups of patients			Analysis			Significant pairwise comparisons
		
	A (N = 67), (%)	B (N = 53), (%)	C (N = 56), (%)	Test value^a^	df	*p *value	
Low	30 (44.8%)	31 (58.5%)	49 (87.5%)	24.2	2	0.000	A > C, B > C
Fair	37 (55.2%)	22 (41.5%)	7 (12.5%)				

^a^Pearson χ^2 ^test.

df = degrees of freedom.

The employment outcome of group C was significantly less satisfactory compared to group A and B (multiple χ^2 ^= 24.2, df = 2, *p *= 0.000), whereas groups A and B did not differ regarding their employment outcome (multiple χ^2 ^test).

Further, to test the effect of age to this finding, we compared the employment outcome of the three groups using the age of 35 years as the cut-off point. The employment outcome of the 26 members of group C aged under 35 years was significantly poorer compared to their 56 counterparts of group A and 43 of group B (multiple χ^2 ^= 14.9, df = 2, *p *= 0.001). In contrast, no differences were found comparing 30 members of group C aged 35 years old or over to their 11 counterparts of group A and to 10 ones of group B (multiple χ^2 ^test).

No differences were observed between males and females with regard to employment outcome in all groups (A: M/F = 43/24, χ^2 ^test, B: M/F = 39/14, χ^2 ^test, C: M/F = 36/20, χ^2 ^test).

With regard to employment status differences between the 2-year period before and after the participation in the prevocational programme in each group (included only full-time and part-time employment), the Cohen's *d *of group A participants was 0.85 ('large') (2.13 (SD 3.0) vs 7.58 (SD 8.5) months), of group B was 0.75 ('large') (1.30 (SD 3.2) vs 6.09 (SD 8.2) months) and of group C was 0.47 ('medium') (0.45 (SD 1.5) vs 2.80 (SD 6.8) months), respectively.

Finally, regarding the clinical outcome, hospitalisation rates throughout the 2-year follow-up period were as follows: 14 members (20.9%) of group A, 5 (9.4%) of group B, and 7 (12.5%) of group C were hospitalised. These differences were not found to be statistically significant (one-way ANOVA test).

## Discussion

To the best of our knowledge, this is the first study that has investigated the diachronic trends in employment outcome of prevocational training for chronic psychiatric outpatients. The results of our study have shown a statistically significant decrease of employment rates during the current decade in comparison with the 1980s among rehabilitees experiencing severe psychiatric disorders.

These findings are in accord with those of Perkins and Rinaldi [2], who reported that unemployment rates among people with longer-term mental health problems increased steadily during the 1990s. Several assumptions could be made for our finding. According to Warner [21] the employment of disabled persons with a psychiatric diagnosis depends on economic growth and overall rate of employment. The national unemployment rates for the age range 18 to 45 during 1987 and 1988 (referring to group A) were 9.9% and 10.4%, respectively; during 1990 and 1991 (referring to group B) were 9.5% and 10.1%, respectively; and during 2002 and 2003 (referring to group C) increased to 12.6% and 12.6%, respectively (Hellenic Statistic Department, personal communication). In our study, the decreased employment rates of the rehabilitees were in the same direction with national unemployment rates. This finding is inconsistent with Perkins and Rinaldi [2], whose research findings showed that the unemployment of chronic psychiatric patients increased despite the decrease of general unemployment in London during the 1990s. In the same vein, Melle *et al*. [22], in Norway, a country with well developed social welfare system and low unemployment rates, followed-up patients with schizophrenia after their hospitalisation and reported that at the end of a 7-year follow-up period their unemployment rates were found to be extremely high (94%). Thus general unemployment should not be considered as the only reason for the unsatisfactory employment outcome of disabled persons with a psychiatric diagnosis.

Structural changes in the state's economy possibly might have restricted the opportunities for unskilled or semiskilled workers to find a job, since more qualifications are now required. The fact that the demand for low skilled or semiskilled jobs has steadily fallen during the last decade in the country might have negative consequences for our rehabilitees. Furthermore, vocational training in the rehabilitation workshops may not provide attendees with adequate supplies to cope with contemporary labour market increasing demands, in terms of advanced educational qualifications and specialised skills, as well as cognitive and social skills. Finally, the disability allowances offered by the state may discourage them to find a part-time low-paid job (the 'benefit trap') [6]. We assume that an interaction of all these adverse factors, namely the national unemployment rates, changes in the job market and the 'benefit trap', contributed to the decreased unemployment rates of the third group.

Furthermore, in order to explore to what extent prevocational training programmes led to an improved employment status, we compared the full-time and part-time employment status of the rehabilitees in the 2-year period before and after their attendance at the rehabilitation program. In all groups the improvement was considerable. However, the rehabilitees of group C improved to a lesser extent than their counterparts of groups A and B. This less satisfactory outcome might have been associated with the obviously poorer employment status of group C rehabilitees before the participation in the programme. We also speculate that additional factors might have contributed to this significant employment decline: in the early 1980s when psychiatric reform started in Greece, mental health professionals were enthusiastic and the Manpower Employment Organization launched an agency for subsidising employers and placing mentally ill people in their enterprises. However, a decade later this endeavour was fading away. Whatever the case, under these circumstances, the prevocational programme of our Department during the current decade (2000s) could not reverse the gloomy perspective of (un)employment for chronic mentally ill persons.

Moreover, the results of our study have shown that the low employment achievements of group C could be imputed to its younger members and not to its older ones, who obviously presented a more chronic course of the disorder. Thus the younger mentally ill trainees were those who might have been more affected by the above-mentioned adversities.

We also searched for any differences between males and females in all groups, and specifically in group C, with respect to employment outcome; however, no differences were observed. Thus both males and females of group C manifested equally less satisfactory employment outcomes compared to the rehabilitees of the other groups.

The finding that hospitalisation rates were similar among the three groups is consistent with an early notion (though partly revised later) that vocational performance and psychiatric symptomatology are unrelated [23,24].

Regarding the limitations of our study it should be mentioned that the first and the third group were natural samples whereas the second was experimental. The latter underwent a considerable attrition within the follow-up period, although the 12 dropouts did not differ from the remaining members in reference to the sociodemographic and clinical characteristics. The lack of a common assessment battery for all three groups is conceived as another limitation of this study.

## Conclusions

Prevocational training in psychiatric rehabilitation is expected to benefit the rehabilitees in many ways, for example, improving personal autonomy, social skills, quality of life, and so on. As far as employment is included as a core measure of outcome, the results of our study indicate that the measured effectiveness of prevocational rehabilitation programs has declined during the current decade. To test the hypothesis that protracted training rehabilitation programs may incubate the 'germ' of institutionalism, we suggest a pilot implementation of the 'supported employment' model and its ensuing evaluation.

## Competing interests

The authors declare that they have no competing interests.

## Authors' contributions

RG wrote the article, supervised the data collection and performed the statistical analysis. ET collected the data, interviewed the patients during the follow-up period and contributed to the writing of the manuscript. MM contributed to the interpretation of the results. NT collected data and contributed to articles' writing. DP contributed to data collection and interpretation of the results. VT designed the study, supervised the data collection and revised it critically for important intellectual content.
